# Clinical Outcomes of Bloodstream Infections in Liver Transplant Recipients: A Ten-Year Single-Center Retrospective Analysis, from Türkiye

**DOI:** 10.3390/antibiotics15010090

**Published:** 2026-01-16

**Authors:** Selda Aydin, Meyha Sahin, Bahadir Ceylan, Tunahan Abali, Safa Arda Akin, Murat Dayangac, Ali Mert

**Affiliations:** 1 Department of Infectious Diseases and Clinical Microbiology, Medical Faculty, Istanbul Medipol University, Istanbul 34080, Turkey; meyha.sahin@medipol.edu.tr (M.S.); bceylan@medipol.edu.tr (B.C.); alimert@medipol.edu.tr (A.M.); 2 İnternational Medical School, Istanbul Medipol University, Istanbul 34214, Turkey; tunahan.abali@std.medipol.edu.tr (T.A.); safa.akin@std.medipol.edu.tr (S.A.A.); 3 Center for Organ Transplantation, Istanbul Medipol University Hospital, Istanbul 34810, Turkey; mdayangac@medipol.edu.tr

**Keywords:** bloodstream infection, carbapenem-resistance Enterobacterales, mortality

## Abstract

**Background/Objectives**: Infections remain a leading cause of morbidity and mortality following liver transplantation, with bloodstream infections (BSIs) representing one of the most critical complications. This study aimed to identify factors associated with mortality in liver transplant recipients who developed BSIs over a 10-year period. **Methods**: This retrospective study was conducted at a tertiary university hospital between 1 April 2014 and 31 December 2024. A total of 467 adult patients underwent liver transplantation during the study period. Among 467 patients, a total of 210 bloodstream infection episodes occurring in 136 patients were included in the study. **Results**: BSIs occurred in 29.1% (136/467) of patients, with a total of 210 episodes. The median age was 55 years (IQR: 45–63). Most transplants (95.2%) were from living donors. Hepatitis B virus infection (27.1%) was the most common underlying etiology of cirrhosis. The majority of BSIs (61.2%) occurred within the first three months post-transplant. A total of 242 pathogens were isolated, with ESBL-producing Enterobacterales identified in 72.6% and carbapenem-resistant Enterobacterales (CRE) in 30.1% of cases. Notably, carbapenem resistance among *Klebsiella* spp. was high at 51.78%. The overall mortality rate was 14.28%. Multivariate analysis identified that a high Pitt Bacteremia Score (hazard ratio [HR] 1.502, 95% confidence interval [CI] 1.361–1.657, *p* < 0.001) and CRE infection (HR 3.644, 95% CI 1.380–9.620, *p* = 0.009) were independent predictors of mortality. **Conclusions**: BSIs are a significant post-transplant complication with high antimicrobial resistance. The Pitt bacteremia score is a strong predictor of mortality and may guide early risk stratification and clinical management in liver transplant recipients.

## 1. Introduction

Liver transplantation (LT) has markedly improved survival and quality of life among patients with end-stage liver disease. Despite these advances, infectious complications—especially during the early post-transplant period and under intense immunosuppressive therapy—remain a leading cause of morbidity and mortality [1,2]. Bloodstream infections (BSIs) are among the most severe infectious complications following LT and represent the leading cause of infection-related mortality in liver transplant recipients (LTRs) [3]. Recent studies over the last decade have reported the incidence of BSIs in LTRs to range from 20.6% to 29.1%, with Gram-negative bacilli (GNB) being the leading pathogens, accounting for 52–58% of cases [4,5]. Mortality rates associated with BSIs vary widely, ranging from 10% to 52%, depending on host-related factors, causative microorganisms, and the appropriateness of antimicrobial therapy [3]. Early diagnosis and prompt initiation of effective antimicrobial treatment are therefore crucial and can be lifesaving in this setting [6].

However, the rising prevalence of antimicrobial resistance in recent years has increasingly complicated the management of BSIs and has been associated with poorer clinical outcomes [3,4]. In particular, BSIs caused by extended-spectrum beta-lactamase (ESBL)-producing microorganisms and carbapenem-resistant Enterobacterales (CRE) have been linked to significantly higher mortality rates among bacteremic patients [7,8]. CRE have emerged as a major clinical concern in BSIs, as they substantially limit therapeutic options and are associated with adverse outcomes [9,10].

This challenge is particularly relevant in Türkiye, which holds the highest rate of living organ donation worldwide and has seen a consistent upward trend in transplant volume, with over 21,000 procedures performed between 2009 and 2024 [11,12]. However, national data also indicate a concerning prevalence of multidrug-resistant pathogens (52.7%) and high infection-related mortality (35.9%) in this population [13,14,15].

Given the high incidence of BSIs, the increasing antimicrobial resistance, and the significant impact of BSIs on survival after LT, it is crucial to understand the factors associated with patient outcomes. Therefore, the aim of this retrospective study was to identify independent predictors of mortality among LTRs who developed BSI at a tertiary care university hospital in Türkiye.

## 2. Results

### 2.1. Characteristics of BSI Episodes

**Figure 1:**
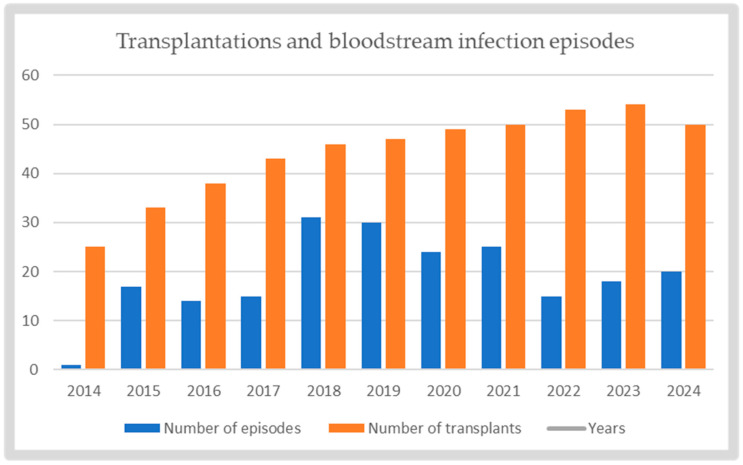
Annual distribution of transplantations performed and BSI episodes.

During the study period, a total of 467 patients underwent LT surgery. BSI occurred in 29.1% (*N* = 136/467) of patients. A total of 210 episodes were documented during the study period. Figure 1 illustrates the annual distribution of transplantations performed and BSI episodes. The overall mortality rate per BSI episode was 14.3% (*N* = 30/210). The median age of patients at the time of BSI episode was 55 years (interquartile range [IQR]: 45–63). The median post-transplant follow-up period was 553 days (IQR: 166–1207), and the median hospital stay following a bacteremia episode was 34 days (IQR: 19.5–51). Table 1 illustrates the demographic, clinical and laboratory characteristics of BSI episodes.

The most prevalent comorbidities were diabetes mellitus (DM) (46.6%), chronic kidney disease (CKD) (15.9%), and coronary artery disease (CAD) (14.4%). A comparison of survivors and non-survivors in terms of comorbidities revealed no statistically significant difference. The most common etiology of cirrhosis was hepatitis B virus (HBV) infection, 27.1%, followed by hepatitis C virus (HCV) infection and metabolic dysfunction-associated steatohepatitis (MASH), 10.5%, and alcohol-related liver disease (ALD), 7.1%. The majority of the LT (95.2%) were performed via living donors. Post-transplant complications were commonly ascites, bile leakage and pneumonia, with percentages of 25.7%, 17.1% and 12.4%, respectively (Table 1).

### 2.2. Source of BSI

Among a total of 210 BSI episodes, the rate of primary bacteremia was identified as 23.8% (*n* = 50). Of all BSI episodes, 61.2% (128/210) occurred within the first three months post-transplant. The most common sources of infection in secondary bacteremia were intra-abdominal infections (52.4%, *n* = 110/210), catheter-related bacteremia (21%, *n* = 44/210), pyelonephritis (2.4%, *n* = 5/210), and pneumonia (1%, *n* = 2/210) (Table 1). Bacteremia secondary to intra-abdominal infections occurred as a consequence of either organ/space surgical site infections or cholangitis. Among catheter-related bloodstream infections, staphylococci were responsible for 43.2% of the causative pathogens, while GNBs accounted for 47.7%. Of the staphylococcal isolates, 76.9% were methicillin-resistant. Prior CRE colonization was identified in 17.6% of patients who developed CRE bacteremia. CRE bacteremia occurred more frequently in colonized patients than in non-colonized patients (26.1% vs. 11.4%); however, the association was not statistically significant (*p* = 0.141).

### 2.3. Microbiological Findings

**Figure 2:**
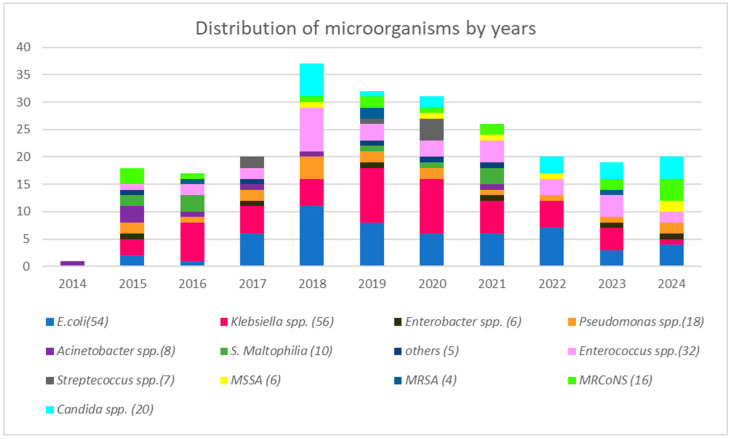
The distribution of microbial agents obtained from blood cultures by year.

Polymicrobial growth was observed in 16.7% (35/210) of BSI episodes. Of these polymicrobial infections, 65.7% comprised either two distinct Enterobacterales species or combinations involving enterococci, while nonfermentative-GNB were present in 28.6% of cases. A total of 242 bacterial and fungal pathogens were isolated from blood cultures. Most common pathogen was *Klebsiella* spp. (23.1%), followed by *E. coli* (22.3%). Among Enterobacterales isolates, 72.6% were Extended-spectrum β-lactamase–producing Enterobacterales (ESBL-E), while 30.1% were carbapenem-resistant Enterobacterales (CRE). The CRE rate was 7.4% (4/54) in *E. coli* isolates and 51.78% (29/56) in *Klebsiella* spp. isolates. Carbapenem resistance was found in 38.8% (7/18) of *Pseudomonas* spp. and in 50% (4/8) of *Acinetobacter* spp. isolates. Among Gram-positive microorganisms, *Enterococcus* spp. was the most frequently isolated species, accounting for 13.2% (32/242) of total isolates. In candidemia cases, the predominant species was *Candida albicans*, with a total of 20 *Candida* spp. isolated. Table 2 summarizes the overall distribution of microbial agents isolated from blood cultures, while Figure 2 details this distribution on a year-by-year basis.

### 2.4. Outcomes

The overall 30-day mortality rate was 14.3% after the onset of a BSI episode. Mortality in patients with CRE bacteremia was 23.5% (*n* = 8/34). Several factors were found to be significantly associated with mortality in LTRs with BSI in the univariate analysis, including cadaveric donor type, a higher Pitt bacteremia score, lower platelet and lymphocyte count, elevated total bilirubin levels, AKI, length of hospital stay and CRE infection (*p* < 0.05). Although early-onset bacteriemia episodes were more likely to have a fatal course, this difference did not reach statistical significance. In contrast, MASH was found to be associated with a high survival rate. However, in multivariable Cox regression model revealed that the Pitt Bacteremia Score (hazard ratio [HR] 1.502, 95% confidence interval [CI] 1.361–1.657, *p* < 0.001) and CRE infection (HR 3.644, 95% CI 1.380–9.620, *p* = 0.009) were independent predictors of mortality. The results of the multivariate analysis of factors associated with mortality are presented in Table 3.

## 3. Discussion

In this single-center retrospective cohort study investigating BSIs after LT, the incidence of BSI was 29.1%, with GNB accounting for 64.8% of isolates. The prevalence of ESBL-E and CRE was notably high, at 72.6% and 30.1%, respectively. The 30-day mortality rate was 14.3%. Among evaluated risk factors, a high Pitt bacteremia score and CRE infection emerged as independent predictors of mortality.

The execution of head-to-head comparisons with earlier studies is difficult because of differences in geographical locations, study periods, and the types of solid organ transplants included. Most published studies have focused on BSI following SOT, regardless of the type of organ transplanted. The incidence rate in our cohort lies within the range reported in previous investigations, which vary between 11.3% and 39.4% among liver transplant recipients [4,5,16,17,18,19,20]. Our incidence was lower than that documented by Yeşilkaya et al. in a national Turkish cohort [5] but higher than the rates reported by Neofytos et al. in the United States and by Adelman et al. in Switzerland [18,20]. Similar infection frequencies have been reported in studies from South Korea (Kim et al.), China (Wan et al.), Italy (Barchiesi et al.), and France (Bert et al.), suggesting a globally consistent burden of post-transplant BSIs [4,16,17,19]. The 14.3% mortality observed in our study also aligns with previously published data, which reported rates between 14.7% and 19.4% [18,20,21,22,23].

Few large-scale studies have focused exclusively on BSIs in liver transplant recipients [4,19,22]. In these large cohorts and other investigations, septic shock, prolonged mechanical ventilation, AKI, and the requirement for hemodialysis following transplantation were identified as major predictors of death [16,17,22,24,25]. We observed that a higher Pitt bacteremia score—reflecting severe infection and the need for intensive care support [26]—was the strongest predictor of mortality, consistent with findings from Santos et al. [22] and Zhang et al. [24]. The present study lends further support to the hypothesis that the Pitt bacteremia score is an effective tool for the assessment of the clinical severity of BSIs.

The global rise in antimicrobial resistance further complicates the management of these infections. According to surveillance data from the Centers for Disease Control and Prevention in the United States and Europe for 2021–2023, the prevalence of CRE varies between 2.8–17.6% and 0–69.7%, respectively [27,28]. Turkish national surveillance reports even higher rates, approaching 55% [29]. In this context, the 30.1% CRE rate observed in our study—particularly the 51.8% resistance rate among *Klebsiella* isolates—represents a serious clinical concern. BSIs due to multidrug-resistant GNB have been consistently associated with limited therapeutic options, increased early mortality, and prolonged hospital stays in transplant populations [30,31,32,33,34]. In the present study, we found that CRE infection significantly increased mortality risk (HR: 3.64, 95% CI: 1.38–9.62). This observation highlights the pressing need for strict infection control measures, early screening, and stewardship programs to mitigate the impact of antimicrobial resistance.

In non-liver transplant populations, the prevalence of CRE among BSIs has been reported to range between 14.3% and 21.6%, with mortality rates of 36.7–56.9% [35,36,37,38]. In contrast, our cohort demonstrated a higher CRE prevalence (30.1%) but a lower mortality rate. Previous studies focusing on solid organ transplant (SOT) recipients have shown that LTRs constitute more than half of CRE BSI cases [34,39]. Besides their increased susceptibility to CRE infections, outcomes in LTRs may be substantially influenced by effective infection control practices and antimicrobial stewardship. Variations in CRE prevalence and mortality reported across studies may reflect multifactorial influences, such as host factors, immunosuppression intensity, clinical severity, comorbidity burden, early diagnosis, source control, and availability of appropriate antimicrobial therapy.

Lymphopenia, an indicator of impaired adaptive immunity, has been linked to worse outcomes in critically ill and post-transplant patients [40]. Although lymphocyte and platelet counts, elevated bilirubin levels, and cadaveric donor type were all associated with mortality in univariate analysis, these variables did not retain statistical significance in multivariable modeling. This discrepancy may reflect differences in patient population, sample size, or the interplay of other dominant risk factors in our cohort.

Despite the varying etiology of BSIs according to study timing and centers, Enterobacterales are the predominant causative agents. In the present study, Enterobacterales were predominant, with a high prevalence of ESBL and CRE production. The relatively lower isolation frequency of non-fermenting GNB in our series suggests that Enterobacterales should remain a primary focus in empirical regimens during the early post-transplant period.

### Limitations of Study

Although the inclusion of a relatively large number of BSI events from a single center and the homogeneity of the study population represent important strengths, it is also essential to acknowledge the inherent limitations of this study. First, its retrospective and single-center design may limit the generalizability of the findings, as local epidemiological patterns, antimicrobial stewardship practices, and transplantation protocols may differ across centers.

Secondly, the retrospective design may have resulted in potential missing data due to incomplete records. Thirdly, the characterization of antimicrobial resistance was based on routine phenotypic antimicrobial susceptibility testing. Specific phenotypic confirmatory assays for ESBL production and for carbapenem resistance mechanisms, as well as molecular analyses to identify underlying resistance genes, were not systematically performed during the study period, which limits the epidemiological interpretation of resistance mechanisms. Finally, as with all retrospective analyses, unmeasured confounders cannot be excluded, and prospective multicenter studies are warranted to validate our findings and clarify independent predictors of mortality in this high-risk population.

## 4. Materials and Methods

### 4.1. Study Design and Setting

This retrospective cohort study included patients who underwent LT between 1 April 2014 and 31 December 2024 at a 970-bed tertiary university hospital in Türkiye with a dedicated solid organ transplantation (SOT) unit. Clinical data were collected through 30 January 2025 to ensure inclusion of all liver transplantation procedures performed up to 31 December 2024 and to allow complete ascertainment of 30-day mortality following BSI onset, with the latest possible BSI onset date of 31 December 2024 and the latest possible death date of 30 January 2025. This study was approved by the Ethics Committee at Istanbul Medipol University (E-10840098-202.3.02-6355; 19 September 2025) with a waiver of informed consent.

### 4.2. Study Population

During the study period, 488 LTs were performed in 467 adult patients (≥18 years), including 21 retransplantations. Among 467 patients, a total of 210 bloodstream infection episodes occurring in 136 patients were included in the study.

Inclusion criteria

- Adult recipients (age ≥ 18 years) who underwent liver transplantation at our center during the study period.
- Availability of core transplant and follow-up data in the electronic medical record.

Exclusion criteria

- Pediatric recipients (<18 years).
- Multi-organ transplantation (e.g., liver–kidney) and/or intestinal/multivisceral transplantation.
- Liver transplants performed outside our center with only follow-up at our institution
- Inadequate documentation to adjudicate BSI status or vital status (e.g., missing transplant date, lack of microbiology data, unknown outcome).

**Figure 3:**
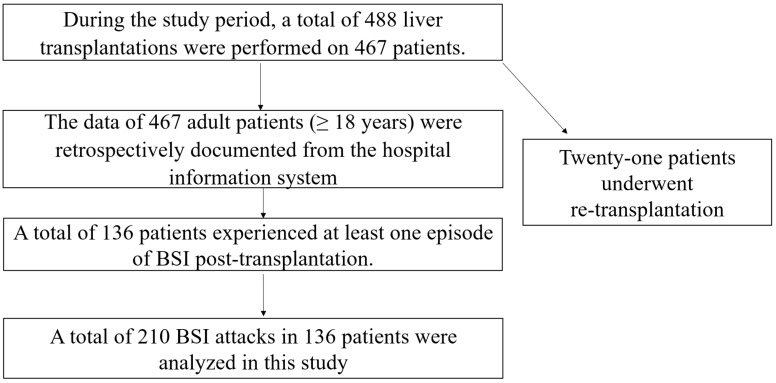
Description of the patient populations included in this study.

The patient inclusion flow chart is summarized in Figure 3.

### 4.3. Data Collection

Patient data were collected from the hospital’s electronic health record system. Demographic characteristics (age, sex), comorbidities [chronic obstructive pulmonary disease (COPD), congestive heart failure (CHF), coronary artery disease (CAD), chronic kidney disease (CKD), diabetes mellitus (DM), and malignancy], etiology of cirrhosis, and donor type (living vs. deceased) were recorded.

Post-transplant complications (pneumonia, ascites, bile leakage, thrombosis), reoperations, and re-transplantations were noted. The timing of BSI onset was classified as early (≤3 months) or late (>3 months) after LT. For each BSI episode, the Pitt bacteremia score, presence of acute kidney injury (AKI), length of hospital stay, and relevant laboratory parameters were documented.

Microbiological data included the microorganisms isolated from blood cultures, antimicrobial susceptibility profiles, and rectal colonization with carbapenem-resistant Enterobacterales (CRE) or vancomycin-resistant enterococci (VRE). The presumed infection source was categorized as intra-abdominal infection, catheter-related BSI, urinary tract infection, pulmonary infection, or primary bacteremia. All-cause mortality within 30 days following bacteremia onset was recorded.

### 4.4. Microbiological Analyses

Blood cultures were routinely obtained from all LTRs with suspected bloodstream infection according to institutional protocols. For each suspected episode, at least two sets of blood cultures (each set consisting of one aerobic and one anaerobic bottle) were collected from separate venipuncture sites prior to the initiation of antimicrobial therapy whenever feasible. Blood culture samples were processed using an automated blood culture system (BacT/ALERT^®^, bioMérieux, Paris, France), and bottles were incubated for up to five days. Positive blood cultures were subcultured on appropriate agar medium, and microbial identification was performed using conventional biochemical methods and/or matrix-assisted laser desorption/ionization time-of-flight mass spectrometry (MALDI-TOF MS; Bruker Daltonics, Bremen, Germany). Antimicrobial susceptibility testing was conducted using automated systems (BD Phoe-nix™, Becton Dickinson, Franklin Lakes, NJ, USA) and/or the disk diffusion method, in accordance with the recommendations of the European Committee on Antimicrobial Susceptibility Testing (EUCAST). Minimum inhibitory concentrations (MICs) were interpreted based on EUCAST clinical breakpoints applicable at the time of isolate recovery [41]. Only microbiologically confirmed bloodstream infection episodes were included in the final analysis.

### 4.5. Definitions

The definition of BSIs is based on the Centers for Disease Control and Prevention/ National Healthcare Safety Network criteria [42].

Primary Bacteremia: Laboratory-confirmed BSI not secondary to any other infection site.

Secondary Bacteremia: BSI secondary to a defined site-specific infection.

Catheter-Related Bacteremia: Positive blood culture in a patient with a central venous catheter in the preceding 48 h and no other identifiable source.

A BSI episode was defined by the presence of at least one positive blood culture bottle containing a recognized pathogenic microorganism. For microorganisms that are potential skin contaminants (e.g., coagulase-negative staphylococci, *Corynebacterium* spp., etc.), the isolation of the same microorganism with similar antimicrobial susceptibility profiles from at least two separate blood culture sets was required to confirm a true infection episode.

Multiple isolates of the same microorganism within 14 days were considered part of a single episode; recurrence after 14 days was regarded as a new episode.

Extended-spectrum β-lactamase–producing Enterobacterales (ESBL-E) were phenotypically identified as isolates resistant to penicillins and oxyimino-β-lactam–containing cephalosporins (including cefuroxime, third- and fourth-generation cephalosporins, and aztreonam), while remaining susceptible to cephamycins and carbapenems. Carbapenem resistance was defined by resistance to at least one carbapenem agent (imipenem, meropenem, or ertapenem) based on EUCAST breakpoints [43].

Carbapenem-non-susceptible *Pseudomonas aeruginosa* (CNSPA): *Pseudomonas aeruginosa* that has tested either intermediate or resistant to at least one of imipenem, meropenem, or doripenem [44].

Carbapenem-non-susceptible *Acinetobacter* spp. (CNSA): Any *Acinetobacter* spp. that has tested either intermediate or resistant to at least one of imipenem, meropenem, or doripenem [44].

### 4.6. Statistical Analysis

Statistical analyses were performed using SPSS version 22.0 software. The normal distribution of variables was assessed using the Shapiro–Wilk test. Descriptive analyses were presented as means ± standard deviations for normally distributed variables, and as medians (interquartile range-IQR) for non-normally distributed variables. Continuous variables were compared using the Mann–Whitney U test due to the violation of parametric test assumptions, while categorical variables were compared using the Chi-square test and Fisher’s Exact test. Thirty-day mortality following BSI was analyzed on a per-episode basis; repeated episodes from the same patient were not adjusted for clustering. Variables potentially associated with mortality were included in the multivariable Cox regression analysis. To avoid model overfitting, the number of covariates included was restricted according to the rule of at least 10 events per variable. Potential collinearity between variables was assessed, and in cases of overlapping effects, clinically relevant variables were prioritized. Results were reported as odds ratios (OR) with 95% confidence intervals (CI), and a two-sided *p* < 0.05 was considered statistically significant.

## 5. Conclusions

BSIs remain a major source of morbidity and mortality after LT. CRE infection and high Pitt bacteremia scores were identified as independent predictors of poor outcomes. Timely diagnosis, prompt initiation of active antimicrobial therapy guided by local resistance patterns, and reinforcement of infection control strategies are crucial for improving survival among liver transplant recipients.

## Figures and Tables

**Table 1:** Demographic and Clinical Characteristics of BSI episodes in Relation to 30-Day Mortality.

Category	Survived *N* = 180 (85.7%)	Died *N* = 30 (14.3%)	Total *N* = 210 (100%)	*p*-Value (>0.05)
**Age (IQR)**	54 (45–63)	57.5 (51–64)	55 (45–63)	0.282
**Sex**				
Male	129 (61.4)	17 (8.1)	146 (69.5)	0.098
Female	51 (24.3)	13 (6.2)	64 (30.5)	
**Comorbidity**				
COPD	3 (1.4)	1 (0.5)	4 (1.9)	0.442
CHF	11 (5.3)	3 (1.4)	14 (6.7)	0.410
DM	82 (39.4)	15 (7.2)	97 (46.6)	0.701
Malignity	26 (12.5)	2 (1)	28 (13.5)	0.384
CAD	24 (11.5)	6 (2.9)	30 (14.4)	0.567
CKD	26 (12.5)	7 (3.4)	33 (15.9)	0.167
**Etiology of cirrhosis**				
HBV	51 (24.3)	6 (2.9)	57 (27.1)	0.400
HCV	19 (9)	3 (1.4)	22 (10.5)	0.640
Alcohol	12 (5.7)	3 (1.4)	15 (7.1)	0.442
MASH	22 (10.5)	0 (0.0)	22 (10.5)	**0.031**
Others	55 (26.2)	11 (5.2)	66 (31.4)	0.703
**Donor type**				
Living	174 (82.9)	26 (12.4)	200 (95.2)	**0.039**
Cadaveric	6 (2.9)	4 (1.9)	10 (4.8)	
**Post-transplant** **complications**				
Pneumonia	21 (10.0)	5 (2.4)	26 (12.4)	0.371
Bile leakage	31 (14.8)	6 (2.9)	37 (17.6)	0.752
Ascites	48 (22.9)	6 (2.9)	54 (25.7)	0.505
Portal vein/hepatic artery thrombosis	15 (7.1)	2 (1.0)	17 (8.1)	1.000
**Timing of bacteremia**				0.069
0–3 months	105 (50.2)	23 (11)	128 (61.2)	
>3 months	75 (35.7)	7 (3.3)	52 (24.8)	
**Pitt bacteremia score**	0 (0–1)	10 (6–12)	0 (0–2)	**0.000**
**Laboratory findings**				
Leukocytes (cell/mm^3^)	6970 (4520–12,190)	6900 (2770–18,000)	6970 (4327–12,777)	0.789
Platelets (×10^3^/mm^3^)	113 (69.5–180.5)	51 (29–104)	105 (60–166.5)	**0.000**
Total bilirubin (mg/dL)	1.53 (0.8–3.25)	5.8 (2.52–11.4)	1.74 (0.89–4.31)	**0.000**
Albumin (g/dL)	3.4 (3.09–3.7)	3.3 (2.85–3.59)	3.4 (3.05–3.7)	0.097
C-reactive protein (mg/L)	111 (57–180)	87.7 (61–142)	107 (57–171)	0.188
Procalcitonin (ng/mL)	4.4 (1.68–50.35)	7.6 (3–22.2)	5.2 (2–35)	0.419
Neutrophil (cell/mm^3^)	5495 (3520–10,245)	5370 (2330–11,200)	5295 (2270–15,550)	0.325
Lymphocyte (cell/mm^3^)	525 (305–830)	290 (190–620)	275 (160–642.5)	**0.011**
**AKI during BSI**	15 (7.2)	12 (5.7)	27 (12.9)	**0.000**
**Repeated surgery intervention**	43 (20.8)	7 (3.4)	50 (24.2)	0.911
**Re-transplantation**	18 (8.6)	3 (1.4)	21 (10.0)	1.000
**Length of stay in hospital (IQR)**	36 (20–55)	24 (17–36)	34 (19.5–51)	**0.038**
**Rectal colonization**				
CRE	20 (10.4)	3 (1.6)	23 (12.1)	1.000
VRE	10 (5.2)	2 (1)	12 (6.3)	0.671
**Polymicrobial BSI**	29 (13.8)	6 (2.9)	35 (16.7)	0.591
**Enterobacteriaceae**				
ESBL	70 (61.9)	12 (10.6)	82 (72.5)	0.488
CRE	26 (23.0)	8 (7.1)	34 (30.1)	**0.035**
**Non-fermentative GNB**				
**CRPA**	4 (26.7)	2 (100)	6 (35.3)	0.110
**CRAB**	2 (50)	3 (75)	5 (62.5)	1.00
**Penicillin resistance**	28 (71.8)	2 (5.1)	30 (76.9)	0.556
**Methicillin resistance**	18 (69.2)	2 (77.7)	20 (76.9)	1.000

IQR: Interquartile range, COPD: Chronic obstructive pulmonary disease, CHF: Congestive heart failure, CAD: Coronary artery disease, CKD: Chronic kidney disease, DM: Diabetes mellitus, HBV: Hepatitis B virus, HCV: Hepatitis C virus, MASH: Non-alcoholic steatohepatitis, AKI: Acute kidney injury, BSI: Bloodstream infection, ESBL: Extended spectrum beta-lactamase, CRE: Carbapenem-resistant Enterobacterales, VRE: Vancomycin-resistant enterococci, GNB: Gram-negative bacilli, CNSPA: Carbapenem-non-susceptible *Pseudomonas aeruginosa*, CNSA: Carbapenem-non-susceptible *Acinetobacter* spp.

**Table 2:** Microorganisms Isolated from Blood Cultures.

Microorganisms (Total: 242)	No (%)
**Enterobacterales**	**121 (50)**
*Klebsiella* spp.	56 (23.1)
*Escherichia coli*	54 (22.3)
*Enterobacter* spp.	6 (2.5)
Other Gram-negative	5 (2.1)
**Non-fermentative GNB**	**36 (14.8)**
*Pseudomonas* spp.	18 (7.4)
*Acinetobacter* spp.	8 (3.3)
*S. maltophilia*	10 (4.1)
**Gram-positive Cocci**	**65 (26.9)**
*Enterococcus* spp.	32 (13.2)
*Streptecoccus* spp.	7 (2.9)
MRCoNS	16 (6.6)
MSSA	6 (2.5)
MRSA	4 (1.7)
**Fungi—*Candida* spp.**	**20 (8.3)**
*C. albicans*	9 (3.7)
*C. galabrata*	6 (2.5)
*C. tropicalis*	2 (0.8)
*C. kefyr*	2 (0.8)
*C. parapsilosis*	1 (0.4)

GNB: Gram-negative bacilli, MRCoNS: Methicillin-resistant Coagulase-negative staphylococci, MSSA: Methicillin-susceptible *Staphylococcus aureus*, MRSA: Methicillin-resistant *Staphylococcus aureus.*

**Table 3:** Multivariate Analysis of Factors Associated with Mortality.

	Hazard Ratio (HR)	Confidence Interval (CI) 95%	*p*
Pitt bacteremia score	1.502	1.361–1.657	0.000
Total bilirubin	1.017	0.967–1.070	0.520
Lymphocyte count	1.000	0.999–1.001	0.458
CRE infection	3.644	1.380–9.620	0.009

## Data Availability

The raw data supporting the conclusions of this article will be made available by the authors upon request.

## Abbreviations

The following abbreviations are used in this manuscript:

LT	Liver transplantation
BSI	Bloodstream infections
LTRs	Liver transplant recipients
GNB	Gram-negative bacilli
IQR	interquartile range
DM	Diabetes mellitus
CKD	Chronic kidney disease
CAD	Coronary artery disease
HBV	Hepatitis B virus
HCV	Hepatitis C virus
MASH	Metabolic dysfunction-associated steatohepatitis
ALD	Alcohol-related liver disease
CRE	Carbapenem-resistant Enterobacterales
VRE	Vancomycin-resistant enterococci
ESBL-E	Extended-spectrum β-lactamase–producing Enterobacterales
HR	Hazard ratio
CI	Confidence interval
COPD	Chronic obstructive pulmonary disease
CHF	Congestive heart failure
AKI	Acute kidney injury
MRCoNS	Methicillin-resistant Coagulase-negative staphylococci
MSSA	Methicillin-susceptible *Staphylococcus aureus*
MRSA	Methicillin-resistant *Staphylococcus aureus*
SOT	Solid organ transplantation
OR	Odds ratios
EUCAST	European Committee on Antimicrobial Susceptibility Testing
CNSPA	Carbapenem-non-susceptible *Pseudomonas aeruginosa*
CNSA	Carbapenem-non-susceptible *Acinetobacter* spp.
